# Editorial: Neurological and Neuroscientific Evidence in Aged COVID-19 Patients

**DOI:** 10.3389/fnagi.2021.774318

**Published:** 2021-10-18

**Authors:** Jennifer A. Frontera, Thomas Wisniewski

**Affiliations:** New York University Grossman School of Medicine, New York, NY, United States

**Keywords:** COVID-19, SARS-CoV-2, aging, dementia, neurodegeneration

The COVID-19 pandemic has touched hundreds of millions of lives worldwide and has had a particularly devastating effect on the elderly, those with pre-existing dementia or neurodegenerative disease and their caregivers. In this special Research Topic of *Frontiers in Aging Neuroscience*, we focus on the epidemiology and mechanisms of new or worsened cognitive impairment following SARS-CoV-2 infection and the implications of post-acute cognitive sequelae of COVID.

In comprehensive reviews, Kalra et al., and Alonso-Bellido et al. outline the spectrum of acute neurological events that have been reported among COVID-19 patients ranging from rare occurrences of acute disseminated encephalomyelitis, myelitis, or Guillain Barre Syndrome and its variants, to more commonly reported events of ischemic or hemorrhagic stroke. In a similar vein, Pavlov et al. report three typical cases of intracerebral hemorrhage following SARS-CoV-2 infection. In a retrospective review of prospectively collected data, Dhillon et al. identified 23/1,243 (2.3%) hospitalized COVID-19 patients with neurological disorders including confusion/delirium, seizures, stroke, movement disorders, peripheral nervous system disorders, and exacerbations of underling neurological conditions, such as multiple sclerosis.

The prevalence of specific acute neurological events appears to vary by age. In a systematic review and meta-analysis, Sullivan and Fischer evaluated 239 articles including 2,307 laboratory confirmed COVID-19 patients, 20% of whom had asymptomatic or mild disease. The most common new neurological events in individuals over age 50 were cerebrovascular events (50%), followed by smell/taste disorders in 24%. In contrast, patients aged 19–50 were most likely to experience smell/taste disorders (78%), with cerebrovascular events occurring in only 11%. Pediatric patients (aged < 19 years) developed smell/taste disorders in 45%, CNS inflammatory disease in 18% and cerebrovascular events in 13%. Similarly, in an observational, retrospective cohort study of 148 COVID-19 patients, Davidescu et al. reported that symptoms of stroke and confusion were more common in those aged ≥ 65 years, while headache was the most common complaint among those <65 years old. Higher death rates were reported in older individuals.

Aside from age, underlying dementia plays a role in the types of neurological complications encountered in COVID-19. Alonso-Lana et al. note in their review that dementia patients often present with atypical COVID symptoms including worsening of baseline confusion/disorientation or functional status or new or worsened behavioral symptoms. Indeed, increased confusion may be the first symptom of COVID-19 among demented patients. In a multicenter, observational, case-control study, Pisaturo et al. matched 23 COVID-19 patients with dementia to 46 non-demented COVID-19 controls who were similar in age, sex, PaO_2_/FiO_2_ ratio and number of comorbidities. Sixty percent of patients with pre-existing dementia exhibited signs and symptoms of delirium, compared to only 2% of non-dementia controls (*P* < 0.001). Dementia patients were more likely to have severe COVID disease and die in-hospital than controls.

In the post-acute setting, neuropsychiatric and cognitive complications of COVID-19 can persist for months. A review by Alonso-Lana et al. highlights the frequent occurrence of persistent post-acute sequelae including anxiety, depression, sleep impairment and post-traumatic stress disorder among survivors of hospitalization for COVID. In a cross-sectional survey of 999 adults across the U.S., 8% reported having COVID (none were hospitalized) and 25% of these patients reported having prolonged COVID-19 symptoms lasting a median of 4 months (range 1–13 months), according to a report by Frontera et al. Among those with a history of long COVID, the most common persistent neuropsychiatric symptoms were anxiety (53%), brain fog/difficulty concentrating/forgetfulness (47%), and headache (47%). However, symptoms of anxiety, headache, brain fog, fatigue, depression, and difficulty sleeping were also reported in >20% of patients *without* a history of COVID. Furthermore, low self-reported scores on NIH Neuro-Quality of Life Metrics for cognition, fatigue, anxiety, depression and sleep occurred in ~30% of COVID-negative respondents, underscoring the contribution of pandemic related stressors to symptomatology. Indeed, 68% of respondents reported at least one socio-economic stressor in the prior month (most commonly social isolation, financial insecurity, fear of illness, and political conflict with family/friends) and 29% of respondents reported serious pandemic-related stressors (including unemployment, financial insecurity, food insecurity, or homelessness). In multivariable analysis, after adjusting for demographics and socio-economic stressors, respondents with a history of COVID still had significantly worse Neuro-QoL scores for subjective cognitive dysfunction compared to COVID-negative respondents, highlighting the relationship between SARS-CoV-2 and protracted cognitive abnormalities. No differences were identified in multivariable analyses for Neuro-QoL scores for anxiety, depression, fatigue, or sleep based on COVID status.

While indirect COVID-19 pandemic-related stressors appear to affect a wide age spectrum of the population, the impacts of social isolation, limited access to medical support systems, and financial strain, are particularly severe for dementia patients and their caregivers even in the absence of SARS-CoV-2 infection. In a survey of 4,913 COVID-negative patients with Alzheimer's dementia, dementia with Lewy bodies, frontal-temporal dementia, and vascular dementia, Rainero et al. report worsening cognitive function in 55% of patients and aggravation of behavioral symptoms in 52% of patients, underscoring the deleterious effects of quarantine. In a review by Hu et al. the authors point out that that pandemic-related safety and logistical issues have compromised diagnostic testing, clinical trial participation and access to therapeutics among those living with neurodegenerative diseases. Similarly, caregivers of dementia patients have reported increased anxiety, depression and distress. In a report by Zucca et al., 90% of caregivers reported at least one symptom of stress and 30% reported four or more symptoms. Risk factors for caregiver burnout were primarily related to relationship conflicts with the patient and pandemic-related interruptions in medical assistance. Compounding these issues, community stressors can lead to sleep deprivation, circadian rhythm disruption and reduced sleep quality, all of which impair immune responses and place older patients and those with underlying sleep disorders, such as obstructive sleep apnea, at higher risk for contracting or dying from SARS-CoV-2, according to a review by Pires et al.

In order to prevent or treat cognitive sequelae from SARS-CoV-2 infection, it is important to understand risk factors and underlying mechanisms of neurovirulence, particularly among vulnerable populations including the elderly and those with underlying neurodegenerative disease. Mainali and Darsie reviewed risk factors for death among older COVID-19 patients and identified pre-existing chronic neurological illness, sleep disturbance, anxiety, depression, immunosenescence, and inflammaging (i.e., increased susceptibility to hyperinflammation with age) as major contributors to COVID severity in the elderly. In a systematic review of neuroimaging and neuropathological findings among older COVID-19 patients (aged > 60 years), Manca et al. noted prominent imaging and pathological evidence of cerebrovascular injury, hypoperfusion, inflammation and cellular damage, particularly along white matter tracts, in the brainstem and frontal-temporal regions. Ganji and Reddy contributed a review addressing the contribution of virus-related mitochondrial injury to disease severity in older individuals. The ACE-2 receptor, which the SARS-CoV-2 virus utilizes for cell entry, is instrumental for mitochondrial function. A paucity of available ACE-2 receptors due to SARS-CoV-2 binding correlates with decreased ATP production. As mitochondria divert to production of radical oxygen species (ROS) in response to viral invasion, this stimulates additional downstream inflammatory responses, cell membrane permeability, and eventual apoptosis. As individuals age, the normal breakdown of mitochondria (mitophagy) occurs less frequently, leading to an accumulation of aged mitochondria that no longer produce energy efficiently. Without mitophagy, levels of ROS rise contributing to oxidative stress, tissue damage and hyperinflammation, which can exacerbate the hyperinflammatory response already present with COVID. Mitochondrial iron storage is also imbalanced in the context of SARS-CoV-2-induced elevated ferritin levels. Raha et al. have further explored the relationship of iron homeostasis and its relationship to SARS-CoV-2 susceptibility. The authors specifically evaluated hepcidin, a hormone involved in systemic iron homeostasis, which also has a role in innate immune responses against viruses. In their study of serum and post-mortem brain samples, they identified co-localized hepcidin and IL-6 in epithelial cells of the choroid plexus, meningeal macrophages and in astrocytes near the endothelium of brain blood vessels of AD and Down's syndrome patients. The authors hypothesized that these patients may be at higher risk for SARS-CoV-2 infection due to imbalanced iron homeostasis and failure of amyloid clearance, leading to neuro-inflammation.

In conclusion, while this topic broadly covers the current COVID-19 literature related to neurological events in the aging and dementia, in this rapidly changing field new discoveries are emerging on a daily basis. The full spectrum of post-acute neurological and cognitive sequelae of COVID-19 and mechanisms of neurological injury are still being elucidated. As our understanding of the neurovirulence of SARS-CoV-2 evolves, it will be important to contextualize the impact of COVID in vulnerable populations, such as the elderly and those with neurodegenerative diseases. Understanding trajectories of cognitive recovery or decline may help guide therapeutic interventions and identify specific targets of pharmacological and rehabilitative interventions.

## Author Contributions

JF drafted the manuscript. TW critically reviewed the manuscript. All authors contributed to the article and approved the submitted version.

## Funding

JF and TW received funding from NIH/NIA grant P30AG066512-01S1 to study neurodegenerative serum biomarkers in hospitalized COVID-19 patients. Dr. Frontera receives funding from NIH/NHLBI 1OT2HL161847-01 and NIH/NINDS 3U24NS11384401S1 for COVID-19 related research.

## Conflict of Interest

The authors declare that the research was conducted in the absence of any commercial or financial relationships that could be construed as a potential conflict of interest.

## Publisher's Note

All claims expressed in this article are solely those of the authors and do not necessarily represent those of their affiliated organizations, or those of the publisher, the editors and the reviewers. Any product that may be evaluated in this article, or claim that may be made by its manufacturer, is not guaranteed or endorsed by the publisher.

